# Pathologies affect the performance of ECG signals compression

**DOI:** 10.1038/s41598-021-89817-w

**Published:** 2021-05-18

**Authors:** Andrea Nemcova, Radovan Smisek, Martin Vitek, Marie Novakova

**Affiliations:** 1 Department of Biomedical Engineering, Faculty of Electrical Engineering and Communication, Brno University of Technology, Technická 12, 616 00 Brno, Czech Republic; 2 Institute of Scientific Instruments, The Czech Academy of Sciences, Královopolská 147, 612 64 Brno, Czech Republic; 3 Department of Physiology, Faculty of Medicine, Masaryk University, Kamenice 753/5, 625 00 Brno, Czech Republic; 4 International Clinical Research Center, St. Anne’s University Hospital Brno, Pekařská 53, 656 91 Brno, Czech Republic

**Keywords:** Cardiology, Biomedical engineering

## Abstract

The performance of ECG signals compression is influenced by many things. However, there is not a single study primarily focused on the possible effects of ECG pathologies on the performance of compression algorithms. This study evaluates whether the pathologies present in ECG signals affect the efficiency and quality of compression. Single-cycle fractal-based compression algorithm and compression algorithm based on combination of wavelet transform and set partitioning in hierarchical trees are used to compress 125 15-leads ECG signals from CSE database. Rhythm and morphology of these signals are newly annotated as physiological or pathological. The compression performance results are statistically evaluated. Using both compression algorithms, physiological signals are compressed with better quality than pathological signals according to 8 and 9 out of 12 quality metrics, respectively. Moreover, it was statistically proven that pathological signals were compressed with lower efficiency than physiological signals. Signals with physiological rhythm and physiological morphology were compressed with the best quality. The worst results reported the group of signals with pathological rhythm and pathological morphology. This study is the first one which deals with effects of ECG pathologies on the performance of compression algorithms. Signal-by-signal rhythm and morphology annotations (physiological/pathological) for the CSE database are newly published.

## Introduction

Electrocardiogram (ECG) is the most frequently used technique to reveal and diagnose heart diseases^1^. In clinical practice, standard 12-lead ECG is used predominantly^2^. Some disorders (especially arrhythmias) are paroxysmal and appear only time to time and/or during specific activity^3^. In such cases, the Holter ECG is indicated^4^. Holter signals are recorded for at least 24 h using mobile ECG device^4^. The obtained signal is stored offline or online transmitted to the medical center and evaluated by cardiologist and/or Holter technician. To speed up the transmission^5^, save the memory^5^ and energy of the device^6^, the ECG signal is compressed.

The aim of compression is to reach maximum efficiency of data reduction without loss of diagnostic information^5^. Compression algorithm may be acceptable only if the diagnostic information (ECG morphology) is neither lost nor distorted^7^. To gain significant data reduction and minimize power consumption in telehealth monitoring, lossy compression is preferred^8^. However, lossy compression is always connected with information loss. As a matter of principle, lossy compression is always compromise between size of the data and their quality^9^. Therefore, the assessment of ECG signal quality after compression and the determination of compression efficiency should be an essential part of compression itself^10^.

In terms of compression algorithms’ approach to pathologies in ECG signal, two groups can be distinguished. First, algorithms which do not take into consideration pathologies in ECG signal, such as Set Partitioning in Hierarchical Trees (SPIHT)^11,12^, compressed sensing based method^13^ or skeleton (local extreme extraction) based method^14^. Second, compression algorithms which take into consideration pathologies in ECG signal (such as^15–18^). The principles of these algorithms are usually based on detection of QRS complexes or beats classification. A few examples of compression algorithms which take into consideration pathologies are described in the next paragraphs in more detail.

Compression methods based on QRS complex detection exhibit lower performance in case of abnormal ECG signals^19^. This problem mainly concerns 2D compression methods in which the ECG signal is always segmented into beats. The changes in beats’ periodicity lower the performance of 2D compression algorithms^7^. The performance of 2D compression methods is dependent on the accuracy of QRS complex detection which may be affected by noise, artifacts, sudden changes in amplitudes, RR intervals or QRS complex morphology^7^. Chen^16^ developed an algorithm based on template matching and stated that it was not very suitable for irregular waveforms, including varying patterns. Chou^17^ developed a 2D ECG preprocessing algorithm for better compression of irregular ECG signals.

Bera et al.^18^ used hybrid compression of ECG signal. It started with support vector machine (SVM)-based binary classifier of ECG beats to normal and abnormal and continued with wavelet-based compression of abnormal beats and combined wavelet- and PCA-based compression of normal beats.

Rakshit et al*.*^15^ used different approach. They precede reconstruction errors by using three dictionaries considering normal, premature ventricular contraction and paced beats to recover the signal in compressed sensing (CS) approach (it is one specific algorithm based on CS; not all CS-based compression algorithms take into consideration pathologies). The ECG morphology differs in these three types of beats and due to the tailored reconstruction can be preserved. This algorithm was tested on a part of MIT-BIH Arrhythmia Database, Normal Sinus Rhythm Database and Compression Test Database. The authors stated that their method outperformed existing methods (wavelet dictionary, adaptive dictionary, standard dictionary based CS approaches and wavelet-based lossy compression scheme). However, this algorithm may have problems with signals including other pathologies.

Nasimi et al*.*^20^ introduced compression scheme which discriminates between normal and abnormal heartbeats. In case of normal heartbeats, the redundancy is removed, which leads to increased sparsity of the signal and such part is better compressible using CS. Dissimilar heartbeats can be caused by pathology and such parts are not compressed. In both cases, the quantization and Huffman encoding are applied.

Compression performance of ECG signal may be decreased by noise, unless it is filtered either before or within the compression process. Noisy ECG signals are usually stored using more bits^7,21^. But noise is not diagnostically valuable; thus it is advantageous not to store it at all (in ideal situation).

PhysioNet contains a database of ECG signals dedicated for testing of compression algorithms—the MIT-BIH ECG Compression Test Database^22,23^. It consists of 168 two-channel ECG signals which include wide variety of pathologies—arrhythmias (atrial, AV junctional, ventricular), disturbances in conduction and noise^23^. It can be used for testing of compression abilities of various algorithms, particularly how they can compress different types of ECG signals and preserve the diagnostic information. Nevertheless, this database is used rather sporadically^5,15,21,24^. The main reason is the fact that it is not annotated^25^.

To the best of our knowledge, there is not a single study primarily focused on the possible effects of ECG pathologies on the performance of compression algorithms. Manikandan et al*.*^7^ reported that the presence of both regular and irregular rhythm with different morphologies in the signals may lead to different compression ratios (CRs) for a given percentage root mean square difference (PRD) in compression methods. Several quality-guaranteed methods are available, e.g.^26–28^, in which the quality metric’s threshold can be set to compress the signal without loss of diagnostic quality. However, this threshold can be set empirically or according to other author’s recommendations which are usually determined on the whole database. In previous study^10^, we recommended various quality metrics’ thresholds. However, if real guarantee of the diagnostic quality of the signal is needed, the quality metrics’ values are very strict in case the whole database is considered. As a result, the efficiency of compression will be low. Potentially, the signals with any pathology need to be compressed more carefully with lower efficiency to reach desired quality. On the other hand, physiological signals can be compressed more efficiently and the quality may be preserved. This study deals with this assumption.

The aims of this study are to evaluate whether the pathologies present in ECG signals affect (a) the quality of the compressed and reconstructed signal and (b) the efficiency of the compression. Algorithms such as SPIHT^11,12^ enable to set the compression efficiency directly and thus they are suitable for solving the first aim. In this case, all signals will be compressed with constant efficiency (average value length = avL) to assess the influence of pathologies on quality. Algorithms such as single-cycle fractal-based (SCyF)^29^ do not enable direct setting of efficiency (avL) or quality after compression. Thus, they are suitable for solving the second aim, where the efficiency (avL) as well as the quality change. The parameters (described in “Methods” section) of the SCyF algorithm (excluding the efficiency and quality ones) will be set equally for all signals. For this study, the ECG signals from the CSE database were used. Firstly, the analysis was performed on signals classified into two groups—physiological and pathological. Secondly, in pathological signals, it was distinguished whether the rhythm and/or morphology is pathological. Thus, more detailed analysis was performed as well. Noise is not considered within this study and it is discussed in limitations.

## Methods

### CSE database and annotations

For the purpose of this study, the second most cited^30^ database—Common Standards for Quantitative Electrocardiography (CSE) database^31^ was used. In this study, only the 125 original (non-artificial) signals from dataset 3 of CSE database were used. These signals are both without and with various pathologies. Each signal was obtained from 15 leads—12 standard leads and 3 Frank leads. The length of each signal is 10 s, sampling frequency is 500 Hz, bit resolution is 16 bps. In our previous study, we freely published the annotations of pathologies for the CSE database^30^. Five cardiologists diagnosed the signals independently and then the 4R consensus was provided by two ECG experts and final diagnoses were determined. Final diagnoses include 38 standard diagnostic statements according to American Heart Association (AHA) recommendations^32^. Details can be found in^30^.

In the present study, 125 signals from the CSE database were classified into 4 groups according to the annotations of pathologies from^30^. Two features were assessed—rhythm and morphology of the signals, each into two classes—physiological (F) and pathological (P); thus, 4 groups were created. Physiological rhythms include following categories: sinus rhythm, short and prolonged PR interval, sinus tachycardia, and sinus bradycardia. Physiological morphology includes normal QRS complexes and supraventricular premature complexes. Pathological rhythms include second-degree and third-degree AV blocks, atrial fibrillation, atrial flutter, and paced rhythm. Pathological morphologies include ventricular hypertrophy, myocardial infarction, premature ventricular complexes, paced complexes, and various disorders of ventricular conduction. Classification was provided by one of ECG experts engaged in the previous study^30^. In Table 1, overview of the groups and the number of signals classified in each group is presented. Table with annotations from our previous article^30^ is within the present study enhanced by rhythm and morphology assessment signal-by-signal. Enhanced table is presented in the Supplementary Table S1.

**Table 1:** Overview of 4 groups of signals according to the presence or absence of pathologies in rhythm and morphology.

Rhythm	Morphology	Group abbreviation	Number of signals
F	F	FF	39
F	P	FP	72
P	F	PF	7
P	P	PP	7

F stands for physiological and P stands for pathological signals.

In case of two-group analysis, three groups with any pathology (FP, PF, PP) were clustered into one group of pathological signals. This group includes 86 signals. The second group includes only FF group consisting of 39 signals.

For testing, all signals from the CSE database were used, only Frank leads of signals no. 60, 68, 76, 84, 92, 100, 108, and 124 were excluded, since they are of constant value.

### Compression and assessment of its efficiency and quality

Compression was provided using two algorithms (a) combination of wavelet transform (WT) and SPIHT^11,12^ and (b) SCyF^29^. In this study, both compression algorithms are lossy ones.

The first algorithm decomposes the signals using WT into wavelet coefficients which create temporal orientation trees. The SPIHT algorithm iteratively codes the coefficients based on their importance using their comparison with threshold. Output of the algorithm is a bit flow. This algorithm can be directly controlled in terms of both efficiency (avL) and quality (normalized PRD = PRDN)^12^. SPIHT algorithm was primarily dedicated to image compression^33^. Later, 1D version of the original 2D SPIHT algorithm was published and applied to ECG^11^. In this study, the SPIHT algorithm implemented by Hrubes et al.^12^, further called SPIHT-H was used.

SCyF algorithm which is based on fractals was introduced in our recent study^29^. SCyF algorithm uses downsampled single-cycle of ECG as a domain. The ECG signals are then divided on range blocks (RB) of block size (BS, in this study BS = 256). For each RB, the most similar domain block (DB, overlapping parts of domain, overlapping is set by jump step = JS which is 1 in this study) is searched. As a similarity metric, the fractal root mean square (FRMS) is used (in this case, FRMS = 12). Similarity between RB and DB can be increased using fractal coefficients and affine transform applied on domain block and/or division of RB (BS is halved, in this case, we used maximally 2 divisions). The outputs of the SCyF compression algorithm are domain, index of DB, fractal coefficients, type of used transform, and number of divisions (all in binary form). This algorithm does not enable direct setting of the quality or the efficiency of compression. Other parameters of the algorithm were set equally as described above.

The first aim was to reveal whether the quality after compression and reconstruction is the same for physiological and pathological signals or whether there are significant differences. For this kind of testing, the SPIHT-H algorithm was used. The efficiency of compression was set equally to avL = 1 bps for all signals.

The second aim was to reveal whether physiological signals are compressed more or less efficiently (avL) and with different quality after compression and reconstruction than pathological signal. In this case, the SCyF algorithm was used.

To assess the quality of the signals after compression and reconstruction, 12 metrics recommended in our previous studies^10,34^ were used. These are namely: percentage similarity using standard deviation of NN (PSim SDNN), quality score (QS), signal to noise ratio (SNR1), mean square error (MSE), normalized percentage root mean square difference (PRDN1), maximum amplitude error (MAX), standard error (STDERR), wavelet-energy based diagnostic distortion using stationary wavelet transform (WEDD SWT), spectra difference (Spectrum), similarity—positions with tolerance of 10 (SiP10), similarity—positions and amplitude with tolerance of 10 (SiPA10), and dynamic time warping—percentage match of fiducial points (DTW pmfp2).

### Statistics

At first, the possible difference between two groups of signals (physiological and pathological) was tested. Normality of the data was tested using Shapiro–Wilk and Lilliefors test. The null hypothesis is that the data come from a normally distributed population. According to these two tests, data were not normally distributed, thus nonparametric test was applied.

Two groups (physiological and pathological) are considered in this case, thus the Mann–Whitney test—a nonparametric test dedicated for two independent groups is chosen. It examines whether these two groups were selected from populations with the same distribution. The null hypothesis is that there is no difference between the groups of physiological and pathological signals.

Secondly, we went deeper and tested whether there is the difference between four groups of signals (FF, FP, PF and PP). Extension of Mann–Whitney test is Kruskal–Wallis test dedicated for two or more independent groups. It examines the null hypothesis whether these four groups originate from the same distribution.

Multiple comparison of mean ranks is used for detail testing after rejecting the null hypothesis in Kruskal–Wallis test. Each pair of groups tested using Kruskal–Wallis test can be compared. Mean and comparison interval of each of 12 metrics in each of 4 groups (FF, FP, PF, and PP) is calculated. The null hypothesis is that the corresponding mean difference between two groups is equal to zero.

## Results and discussion

### SPIHT-H (avL = 1 bps)

#### Two groups

In Table 2, mean and median quality is expressed by 12 quality metrics for groups of physiological and pathological signals from CSE database. It was compared which of groups shows higher quality after compression and reconstruction while the efficiency is constant. Such group is highlighted in green. In most cases, better quality is reached in group containing physiological signals. According to QS, the mean and median qualities are better in pathological group. Median PRDN, SNR and WEDD SWT show also better quality in pathological group. Although in these 5 cases, the pathological signals are of better quality than physiological signals, the difference between the quality metrics is very small. On the other hand, if the physiological signals are of better quality, the difference is in most cases high.

**Table 2:**
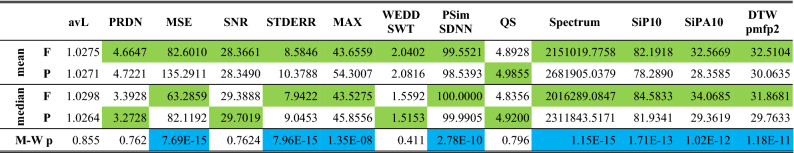
Evaluation of the differences between groups of physiological (F) and pathological (P) signals from CSE database compressed by SPIHT-H method.

Mean and median qualities are expressed by 12 quality metrics. Green color highlights better results. M–W p means p value of Mann–Whitney test. Blue color stands for rejection of null hypothesis.

Statistical analysis was performed on these data as well. As described in “Methods”, Shapiro Wilk and Lilliefors test were used to test normality of data. As both tests showed that the data were not normally distributed, nonparametric Mann–Whitney (M–W) test was used for testing whether there was a difference between physiological and pathological groups. The p value of M–W test is shown in Table 2 in which the cases when the null hypothesis was rejected (p < 0.05) are highlighted in blue. The M–W p values correspond with median. In case the physiological signals show better results in terms of median, the null hypothesis of M–W test was rejected. In case the physiological signals show worse results according to median, the null-hypothesis of M–W is not rejected.

#### Four groups

In Table 3, mean and median results for four groups of signals (FF, FP, PF, and PP) are expressed by 12 quality metrics and one efficiency metric (avL). The best results (the best quality of signals after compression and reconstruction) are highlighted in green and the worst ones in red. In 8 cases out of 12, the best mean results are reached in group FF. In 4 cases, group FP shows the best results. On the other hand, the worst results are found in group PP (8 out of 12 metrics). Three worst results are shown also in group PF and one in group FP. For median, the results are similar. In 8 out of 12 cases, the best results are in group FF. In four cases, the FP group shows the best results. The worst results are in groups PP and PF in 8 and 4 cases, respectively. It is obvious that the group of signals with physiological rhythm and physiological morphology can be generally compressed with the lowest error. According to most of quality metrics, signals with physiological rhythm and pathological morphology are compressed with lower error than signals with pathological rhythm and physiological morphology. Finally, the signals with pathological rhythm and pathological morphology are compressed with the highest error (the worst quality).

**Table 3:**
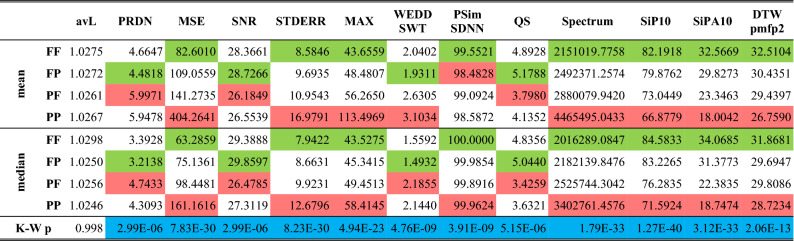
Evaluation of the differences between four groups of signals from CSE database, namely: physiological rhythm and physiological morphology (FF), physiological rhythm and pathological morphology (FP), pathological rhythm and physiological morphology (PF), pathological rhythm and pathological morphology (PP).

Mean and median quality are expressed by 12 quality metrics. Green color highlights the best results and red color highlights the worst results. K–W p means p value of Kruskal–Wallis test. Blue color stands for rejection of null hypothesis.

**Figure 1:**
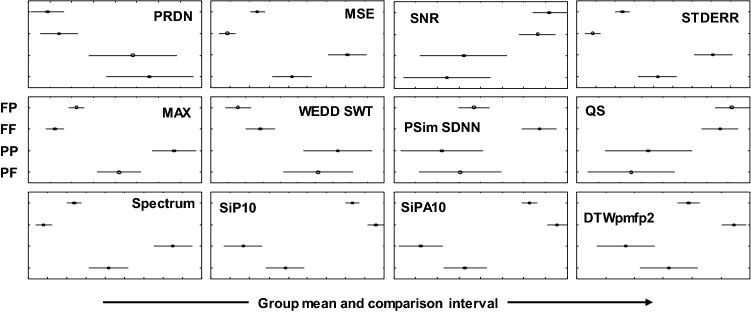
Graphs of multiple comparison of mean ranks.

The null hypothesis whether all four groups originate from the same distribution was tested. For this purpose, non-parametric Kruskal–Wallis test was used. The Kruskal–Wallis p value is shown in Table 3. Blue color highlights the rejection of null hypothesis (p < 0.05) in all cases. It means that not all groups originate from the same distribution. Thus, further testing was performed using multiple comparison of mean ranks (group mean ranks) to compare each pair of groups (Fig. 1). The results (p values) are summarized in Table 4.

**Table 4:**
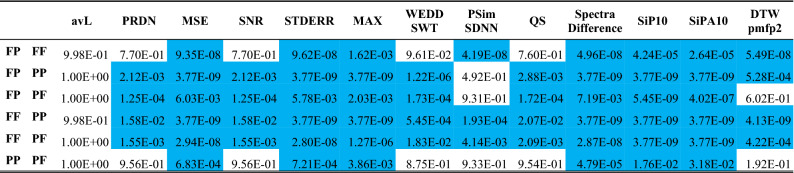
The results of multiple comparison of mean ranks of four groups of signals from the CSE database.

Rejection of null hypothesis is highlighted in blue.

In Table 4, the rejection of null hypothesis is highlighted in blue. The rejection of null hypothesis corresponds with disjoint of intervals in Fig. 1. It means that most pairs of groups differ significantly. The difference between the groups is illustrated by Fig. 1. The difference between FF and PP groups is statistically significant according to all 12 quality metrics. FF and PF groups also differ according to all quality metrics, FF and FP groups differ according to 8 metrics. PP and PF groups differ according to 6 quality metrics. From Table 3 and Fig. 1, it is evident that signals with physiological rhythm and pathological morphology are compressed with lower error than signals with pathological rhythm and physiological morphology in a statistically significant manner (in 10 cases). According to metric PSim SDNN we cannot reject the null hypothesis in half of pairs of groups. It can be stated to that this metric is the least dependent on presence/absence of pathologies in the ECG signal.

### SCyF

#### Two groups

Table 5 shows the results of testing the SCyF compression algorithm on two groups of signals (physiological and pathological). The SCyF algorithm was set equally for all signals. Thus, the effect of pathology presence on compression efficiency (avL) can be evaluated. It is evident that the group of physiological signals is compressed more effectively (with lower avL, highlighted in yellow) than the group of pathological signals. The difference is statistically significant (highlighted in orange). The mean and the median qualities of the reconstructed signals are also better in the group of physiological signals according to 11 out of 12 metrics (except for WEDD SWT). In 9 out of the previously mentioned 11 metrics, the difference is statistically significant (highlighted in blue).

**Table 5:**
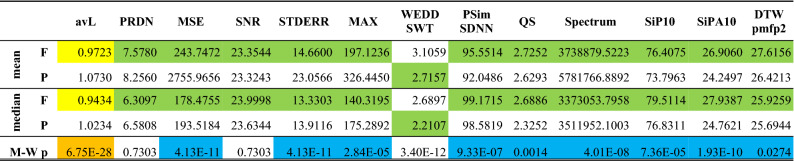
Evaluation of the differences between groups of physiological (F) and pathological (P) signals from the CSE database compressed by the SCyF algorithm. Mean and median qualities are expressed by 12 quality metrics. Yellow color highlights better result in terms of compression efficiency. Green color highlights better results in terms of the quality of signals after compression and reconstruction. M-W p means p-value of Mann-Whitney test. Orange color stands for rejection of null hypothesis in case of avL. Blue color stands for rejection of null hypothesis in case of the quality metrics.

Lossy compression is generally trade-off between its efficiency and quality of the signals after compression and reconstruction. If the avL would be set equally for each signal (as in case of SPIHT-H algorithm), the quality difference between the group of physiological signals and the group of pathological signals would be higher and even more quality metrics would be better for physiological signals.

#### Four groups

The testing of compression performance on signals from 4 groups was performed by SCyF algorithm. The results are shown in Table 6. At first, Kruskal–Wallis test was used to examine whether all four groups originate from the same distribution. The null hypothesis was rejected in all cases (p < 0.05, blue color in the second line of Table 6) and thus further testing using multiple comparison of mean ranks was performed.

**Table 6:**
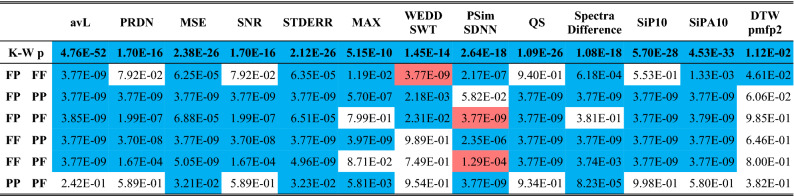
The second line shows the results (p-values) of Kruskal–Wallis test to examine whether all four groups originate from the same distribution.

The rest of the table shows the results of multiple comparison of mean ranks to reveal whether each pair of groups differ from each other in a statistically significant way. Rejection of null hypothesis is highlighted in blue and red. The red color means that the quality of the signal after compression and reconstruction is better for the other group than it was assumed.

The assumption in case of comparing of FF with any other group is that FF signals show better quality after compression and reconstruction. In case of comparison of PP with any other group, the assumption is that PP signals show worse quality after compression and reconstruction. From previous results (using SPIHT-H algorithm) we also know that signals with pathological rhythm (PF) are compressed with worse quality than signals with pathological morphology (FP). The same we assume in case of using SCyF algorithm. Multiple comparison of mean ranks shows that pairs of groups FP and FF, FP and PP, FP and PF, FF and PP, FF and PF differ from each other significantly according to the majority of quality metrics (blue and red color in Table 6). The blue color means that the quality is better for the group according to assumption. If the assumption is not fulfilled, the result is highlighted in red.

It confirms the theory that pathological signals are compressed with lower performance than physiological signals. Moreover, the efficiency of compression (avL value) differs between the groups (except for comparison of PP and PF) significantly as well which can be seen in the avL column in Table 6. If the avL would be the same for FF, FP, PF, and PP groups, the differences in quality would be even higher because lossy compression is a compromise between compression efficiency and signal quality.

### Analysis of pathological signals compression

**Figure 2:**
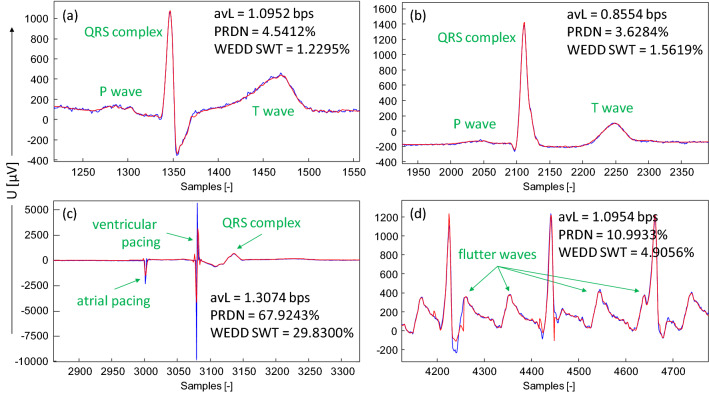
An example of compression of ECG signals without and with pathologies in rhythm and/or in morphology. The SCyF algorithm was used for compression. The blue color stands for the original signal and the red one for compressed and reconstructed signal. Diagnoses are marked in green. (**a**) Normal ECG and sinus tachycardia; physiological rhythm and physiological morphology, lead V2 of signal 16; (**b**) normal ECG and sinus rhythm; physiological rhythm and physiological morphology, lead V3 of signal 73; (**c**) AV dual-paced complexes or rhythm; pathological rhythm and pathological morphology, lead aVF of signal 70; (**d**) atrial flutter; pathological rhythm and physiological morphology, the 2nd Frank lead of signal 111.

The analysis of pathological signals compression was performed using the SCyF algorithm to reveal particular differences in compression of physiological signals and signals with pathologies. Original signal is colored in blue, compressed and reconstructed signal in red. The efficiency of compression in terms of avL and the quality after compression and reconstruction in terms of the most common PRDN and advanced WEDD SWT metrics are shown in Fig. 2.

In Fig. 2a,b, examples of compression of two signals with physiological rhythm and physiological morphology are presented. These signals are compressed with quite low avL (high efficiency) and low distortion (PRDN, SWT WEDD).

Figure 2c shows the detail of signal no. 70 (lead aVF) with a pacemaker peak. This signal belongs into PP group (pathological rhythm and pathological morphology). Pacemaker peak is sharp and it also includes high-frequency components. The results of compression using SCyF algorithm shows significant distortion and higher avL (lower efficiency). In this case, it is caused by the setting of the SCyF algorithm in which the downsampling of the domain is used and thus the sharp pacemaker peak cannot be preserved. This problem can be solved by different setting of the algorithm (no downsampling of the domain) at the cost of the efficiency reduction (avL = 1.8756 bps, PRDN = 4.1994%, WEDD SWT = 3.0441%).

Figure 2d shows the detail of signal no. 111 (the 2nd Frank lead) with pathological rhythm and physiological morphology. The signal was diagnosed as an atrial flutter. The distortion may be caused by irregular occurrence of flutter waves and their transition to QRS complex. In SCyF compression algorithm, as the domain only single ECG cycle is used and thus the algorithm may have problem with cycles that look differently.

### Limitations of the study

The influence of pathologies on compression performance was tested only on one standard database (CSE database). Although this database includes various pathologies, it does not include all usual types (e.g. ventricular tachycardia, ventricular fibrillation or idioventricular rhythm). On the other hand, each signal of this database is annotated. Five cardiologists diagnosed each signal and thereafter two ECG experts made 4R consensus. This process was very time- and source-consuming and it would be very difficult to repeat it for any other database.

Although signal compression is influenced by noise, this effect has not been studied. It is a very complex problem, which is out-of-scope of this study. It would deserve a separate study. The original signals from standard CSE database were used in this study. These signals were not filtered. The reason is that the filtration puts errors in the signals and the analysis would not be objective. Although both used compression algorithms have filtration property, to reach the objective results of compression performance, the clear signal (without noise) should be known which is not possible under real conditions. The solution would be to create artificial signals with different pathologies and known level of noise.

Since in this study only two compression algorithms were employed, its results cannot be generalized on all existing compression algorithms. On the other hand, although both used algorithms are based on different principles, their outcome is very similar. In another words, both algorithms used in this study show the same trend—physiological signals are compressed better than pathological signals.

## Conclusion

As stated in “Introduction”—to the best of our knowledge, there is not a single study primarily focused on the possible effect of ECG pathologies on the performance of compression algorithms. The aims of this study were to evaluate whether the pathologies present in ECG signals affect (a) the quality of the compressed and reconstructed signal and (b) the efficiency of the compression.

According to this study results, significant differences exist between compression of physiological signals and signals with various rhythm and/or morphology pathologies. The differences were proven based on two compression algorithms of different principles (SPIHT-H and SCyF) and majority of 12 quality metrics. The differences vary among quality metrics which supports our previous recommendation to use a combination of several methods for the robust assessment of ECG signal quality after compression^10^.

While the efficiency of compression is constant (SPIHT-H algorithm was used), it was proven that pathological signals were compressed with lower quality than physiological signals according to the majority of used quality metrics. The more detailed analysis of four groups of signals showed that signals with physiological rhythm and physiological morphology were compressed with the best quality according to majority of quality metrics. The worst results reported the group of signals with pathological rhythm and pathological morphology. The difference between these two groups was significant according to all 12 quality metrics. Signals with physiological rhythm and pathological morphology were in most cases compressed with lower error than signals with pathological rhythm and physiological morphology. It was observed that PSim SDNN quality metric was the least dependent on the presence of pathologies.

It was statistically proven that pathological signals were compressed with lower efficiency than physiological signals using the same setting of SCyF algorithm for all signals. Although, compression is always a trade-off between efficiency and quality of compression and in our study the efficiency was lower for pathological signals, the quality was also lower for pathological signals according to majority of quality metrics. More detailed analysis of four groups revealed very similar conclusions as the same analysis using SPIHT-H algorithm (described above). Thus, it is evident that pathologies affect the performance of compression algorithms.

This study is the first one of its kind and brings new knowledge into the area of compression. Moreover, it enhances the annotations of the CSE database in terms of classification the signals into physiological and pathological ones from two points of view—rhythm and morphology. The enhanced annotations can be used in broader area of ECG signal processing and are not limited to compression.

## Supplementary Information

Supplementary Information 1.

## Data Availability

CSE database analyzed in the current study are available from prof. Paul Rubel, INSERM ERM107-MTIC Hôpital Cardiologique, 28 avenue du Doyen Lépine, 69677 BRON Cedex, France but restrictions apply to the availability of these data, which were used under license for the current study, and so are not publicly available. Data are however available from the authors upon reasonable request and with permission of prof. Paul Rubel, INSERM ERM107-MTIC Hôpital Cardiologique, 28 avenue du Doyen Lépine, 69677 BRON Cedex, France.
