# Supplementary material for: Pathologies affect the performance of ECG signals compression
Source: Sci Rep. 2021 May 18;11:10514. doi: 10.1038/s41598-021-89817-w (PMC8131635; doi:10.1038/s41598-021-89817-w)
Supplement: Supplementary file 1 — Supplementary Information 1. [file 41598_2021_89817_MOESM1_ESM.pdf]

Pathologies Affect the Performance of ECG Signals Compression

Andrea Nemcova[1*], Radovan Smisek[1,2], Martin Vitek[1], and Marie Novakova[3,4]

[1]Department of Biomedical Engineering, Faculty of Electrical Engineering and Communication, Brno University of Technology, Technická 12, 616 00 Brno, Czech Republic
[2]Institute of Scientific Instruments, The Czech Academy of Sciences, Královopolská 147, 612 64 Brno, Czech Republic
[3]Department of Physiology, Faculty of Medicine, Masaryk University, Kamenice 753/5, 625 00 Brno, Czech Republic
[4]International Clinical Research Center, St. Anne's University Hospital Brno, Pekařská 53, 656 91 Brno, Czech Republic

*Corresponding author:
Andrea Nemcova
nemcovaa@vutbr.cz

Supplementary material

Supplementary Table S1

Table S1: Table with CSE database annotations from our previous article [30] enhanced by rhythm and morphology assessment signal-by-signal (highlighted in red). Rhythm as well as morphology are each classified into two groups – physiological/pathological. Rhythm: F = no serious pathology present (sinus rhythm, tachycardia and bradycardia); P = pathological. Morphology: F = without morphology changes; P = pathological.

Record	Cardiologist					Consensus	Rhythm	Morphology
	1	2	3	4	5			
**1**	20;143	20;106	20;101;106	20;101;105	20;101;105	**20;101;105**	F	P
**2**	21;226	21;226	21;226	21;142	20;165;226	**21;226**	F	P
**3**	1;21	1;20	21;101	21;101	20;101	**21;101**	F	P
**4**	1;20	1;20	1;20	1;20	1;20	**1;20**	F	F
**5**	21;143;163	20;143	1;21	20;143	20;143	**20;143**	F	P
**6**	20;36	20;30	20;30	20;36	20;36	**20;36**	F	F
**7**	21;142	1;21	1;21	20;142	21;142	**21;142**	F	P
**8**	1;20	1;20	1;20	1;20	1;20	**1;20**	F	F
**9**	20;106	20;106	20;106	20;106	20;106	**20;106**	F	P
**10**	22;63;88	20;88;106	63;88	88;106	88;106	**88;106**	P	P
**11**	1;20	1;20;30	1;20;30	1;20;30	1;20;30	**1;20;30**	F	F
12	*20;142*	*20;101;160;163*	*20;82;226*	*20;226*	*20;82;101*	***20***	F	F
**13**	20;173	20;165	20;165	20;165	20;165	**20;165**	F	P
**14**	20;106	20;106	20;106	20;106	20;106	**20;106**	F	F
**15**	1;22	1;22	1;22	1;22	22;108	**1;22**	F	F
**16**	20;143	1;21	1;21	1;21	1;21	**1;21**	F	F
**17**	20;101	20;104	20;104	20;101	20;104	**20;104**	F	P
**18**	50	50	50	50	50	**50**	P	F
**19**	20;162	1;20	1;20	1;20	1;20	**1;20**	F	F
**20**	21;165	21;36;160	21;36;142;165	21;165	21;30;142;160	**21;36;165**	F	P
**21**	22;105	20;105	1;22	22;105	20;105	**22;105**	F	P
**22**	20;82;165	20;82;160	20;82;160	20;82;165	20;82;165	**20;82;165**	F	P
**23**	22;84;107	20;25;30	22;30	20;25;30	22;30	**22;25;30**	F	F
**24**	20;104	20;104	20;104	20;104	20;104	**20;104**	F	P
**25**	1;20	20;80	1;20	20;80;163	20;80	**20;80**	F	F
**26**	20;60;101;106	20;60;82; 101;106	20;60;101;106	20;101;106	20;60;101;106	**20;60;101;106**	F	P
**27**	20;143	1;20	20;143	20;105	21;143	**20;143**	F	P
**28**	20;66;142	20;60;61;142	20;142	20;60;142;345	20;60;142	**20;60;142**	F	P
**29**	20;106	20;106	20;106	20;106	20;106	**20;106**	F	P
**30**	20;142	20;142	20;142	20;142	20;142;226	**20;142**	F	P
**31**	20;162	1;20	20;162	20;143;162	20;162	**20;162**	F	P
**32**	23	21;23	23;165	23;165	20;30;165	**23;165**	P	P
**33**	20;101;106	20;101;106	20;101;106	20;101;106	20;101;106	**20;101;106**	F	P
**34**	20;60	20;60	20;60;342	20;60;342	20;60	**20;60;342**	F	P
**35**	20;155	1;20	1;20	20;155	20;155	**20;155**	F	P
**36**	20;162	1;20	20;162	20;162	20;162	**20;162**	F	P
**37**	20;30	23	20;30	1;20	20;23;30	**20;30**	F	F
**38**	20;155	1;20	1;20	1;20	20;155	**1;20**	F	F
**39**	22;163	20;166	20;22;165	20;160	20;166	**20;166**	F	P
**40**	20;38;60;101	20;60;101	20;60;101;142	20;60;101;142	20;60;80;142	**20;60;101;142**	F	P

**41**	20;106	20;106	20;106	20;105	20;106	**20;106**	F	P
**42**	1;20	1;20	20;105	20;165	20;226	**20**	F	F
**43**	20;155	20;155	1;20	20;155	20;155	**20;155**	F	P
**44**	20;155	20;155	20;22	20;162	20;155	**20;155**	F	P
**45**	50;106	50;102;106	38;143;160	38;102;106	50;102;106	**50;102;106**	P	P
**46**	20;104	20;104	20;104	20;104	20;104	**20;104**	F	P
47	*20;36;143*	*20;30*	*21;30;165*	*20;102;165*	*21;30;143*	***20;30***	F	F
**48**	20;101	20;82	20;142;143;165	20;101;109	20;82;101;165	**20;101**	F	F
**49**	20;142;162	1;20	20;142;162	20;161	20;162;163	**20;162**	F	P
**50**	20;108	20;108	20;38;106	20;108	20;108	**20;108**	F	P
**51**	21;143	21;143	21;143	1;21	20;143	**21;143**	F	P
52	*50;101*	*50;125*	*50;162*	*50;162*	*50;101*	***50***	P	F
**53**	21;143	21;143	20;143	20;143	21;143	**21;143**	F	P
**54**	20;60;83;<br>162	20;89;161	20;60;83;162	20;60;83;162	20;83;160	**20;60;83;162**	F	P
**55**	20;155	20;155	1;20	1;20	20;155	**20;155**	F	P
**56**	20;200	20;125	20;125	20;125	20;125;200	**20;125**	F	F
**57**	50	51	50;51	50	50;51	**50;51**	P	F
**58**	20;80	20;80	1;20	20;80	80	**20;80**	F	F
**59**	20;155	20;155	1;22	1;20	20;155	**20;155**	F	P
**60**	20;155	1;20	1;20	1;20	1;20	**1;20**	F	F
**61**	20;60;82	20;60;82	20;60;82;105	20;60	20;60;82	**20;60;82**	F	F
**62**	1;20	1;20	1;20	1;20	1;20	**1;20**	F	F
**63**	20;230	20;230	20;143	20;143	20;230	**20;230**	F	F
64	*20;155;214*	*20;165*	*1;20*	*20;142*	*20;155;165;214*	***20***	F	F
**65**	20;60;104	20;60;104	20;60;104	20;30;104	20;60;104	**20;60;104**	F	P
**66**	20;155	20;160	20;162	1;20	20;162	**20;162**	F	P
**67**	181	181	181	181	181	**181**	P	P
**68**	21;162;165	21;160	21;162;165	20;165	21;160	**21;165**	F	P
**69**	20;162	20;163	20;162	20;161	20;163	**20;162;163**	F	P
**70**	184	184	184	184	184	**184**	P	P
**71**	20;155	1;20	1;20	20;155	20;155	**20;155**	F	P
**72**	20;155	1;20	1;20	20;145;363	1;20	**1;20**	F	F
**73**	1;20	1;20	20;107	1;21	1;20	**1;20**	F	F
**74**	20;60;101;106	20;60;82;106	20;60;101;106	20;60;101;105	20;60;82;106	**20;60;101;106**	F	P
**75**	20;60;345	20;60;345	20;60;345	20;60;345	1;20	**20;60;345**	F	P
**76**	20;101;106	20;101;106	20;101;106	20;101;106	21;101;106	**20;101;106**	F	P
**77**	1;20	1;20	20;155	1;20	1;20	**1;20**	F	F
**78**	1;20	1;21	1;22	1;22	1;20	**1;22**	F	F
79	*1;20*	*20;142*	*20;205;226*	*20;142*	*20;145;226;362*	***20***	F	F
**80**	1;20	20;160;163	20;142;163	20;163	20;162;163	**20;163**	F	P
**81**	20;165	20;165	20;101;160	20;165	20;165	**20;165**	F	P
**82**	1;20	20;25	25	20;25	20;25	**20;25**	F	F
**83**	1;20	20;143	20;143	20;143	20;143	**20;143**	F	P
84	*20;160*	*20;106;166*	*20;106;160;163*	*20;105;165*	*20;105;160*	***20;160***	F	P
**85**	20;101	20;104	20;104	20;104	20;104	**20;104**	F	P
**86**	20;163	20;26;101;160;163	20;101;163	20;101;160	20;101;163	**20;101;163**	F	P
**87**	1;20	20;26;142;226	20;226	20;142	20;21	**20;142;226**	F	P
**88**	1;20	20;26	1;20	1;20	1;20	**1;20**	F	F
**89**	50	26;50	42;50	50	50	**50**	P	F
**90**	20;165	20;26;101;165	20;165	20;101;165	20;165	**20;165**	F	P

**91**	20;163	20;26;162;163	20;142;162	20;162;163	20;163	**20;162;163**	F	P
**92**	50;142;226	26;50;142;226	50;205	50;142	50;142;226	**50;142;226**	P	P
**93**	50	26;50	50	50	50	**50**	P	F
**94**	82	20;26;82;226	21;82;83	20;82;226	89;142	**20;82;226**	F	P
**95**	1;20	20;62;142;226	20;162;205	20;142	23;226	**20;142;226**	F	P
**96**	20;163	20;26;82;160	20;82;143	20;82;160;1 63	20;82;163	**20;82;163**	F	P
**97**	1;20	20;26	20;155	1;20	20;82	**1;20**	F	F
**98**	20;165	20;104	20;108	20;104	20;104	**20;104**	F	P
**99**	1;20	1;20	1;20	1;20	1;20	**1;20**	F	F
**100**	50;51	51	50	51	50	**50;51**	P	F
**101**	21;165	1;20	1;21	1;20	1;21	**1;21**	F	F
**102**	20;131; 140;162	1;20	1;21	1;20	1;21	**1;20**	F	F
**103**	20;36;60; 105;345	20;30;60;345	20;60;101	20;30;60; 101;345	20;30;60	**20;30;60;345**	F	P
**104**	20;163	1;21	20;160	20;162;165	20;160	**20;160**	F	P
**105**	20;60;101; 106;345	20;60;106;345	20;60;101;10 6	20;60;101; 106;345	21;30;106	**20;60;101;106;345**	F	P
**106**	1;20	1;20	1;20	1;20	1;20	**1;20**	F	F
**107**	20;104	20;104	20;104	20;104	20;104	**20;104**	F	P
**108**	20;165	20;165	20;160;163	20;165	20;160	**20;165**	F	P
**109**	23;60; 165;345	23;60;102;345	20;30;60;104	20;60;160; 163;345	20;60;102	**20;60;102;345**	F	P
**110**	20;165	20;160	20;160;163	20;160;163	20;160	**20;160**	F	P
**111**	51;105	51	51;105	51	51	**51**	P	F
**112**	20;60;160; 163;345	20;60;160; 163;345	20;60;160;16 3	20;60;160; 163;345	20;60;160	**20;60;160; 163;345**	F	P
**113**	20;105	20;105	1;21	20;105	20;105	**20;105**	F	P
**114**	20;160;162	20;160	20;101;162;1 63	20;163	20;162;163	**20;162;163**	F	P
**115**	20;60;61	21;60;345	21;60	21;60	21;60	**21;60**	F	F
**116**	1;20	1;20	1;20	1;20	1;20	**1;20**	F	F
**117**	23;101;104; 142;341;345	20;60;101; 142;341	20;60;101;10 4; 160;341	20;60;101; 104;341	20;60;101;14 2; 341;345	**20;60;101;104; 142;341**	F	P
**118**	20;107;145; 161;363	20;160;163	20;163	20;161	20;160;163	**20;163**	F	P
**119**	20;82;142	21;82;142	20;82;142;16 3	21;82;142	21;82;142	**21;82;142**	F	P
**120**	42;50;60;106	50;60;101;106	42;50;60;101 ;106	50;106	50;60;101;10 6	**50;60;101;106**	P	P
**121**	25;162	20;25;37;162	20;25;162	20;84;162	20;25;162	**20;25;162**	F	P
**122**	20;66;165	20;60;165	20;60;165	20;60;101; 165;345	20;60;101;16 5	**20;60;165**	F	P
**123**	20;101	20;106	20;106	20;105	20;106	**20;106**	F	P
**124**	1;20	1;20	1;20	1;20	1;20	**1;20**	F	F
**125**	1;20	1;20	1;20	1;20	1;20	**1;20**	F	F
